# Supplementary material for: The Fungal Root Endophyte Serendipita indica (Piriformospora indica) Enhances Bread and Durum Wheat Performance under Boron Toxicity at Both Vegetative and Generative Stages of Development through Mechanisms Unrelated to Mineral Homeostasis
Source: Biology (Basel). 2023 Aug 7;12(8):1098. doi: 10.3390/biology12081098 (PMC10452518; doi:10.3390/biology12081098)
Supplement: Supplementary file 1 [file biology-12-01098-s001.zip › Fig. S1.pdf]

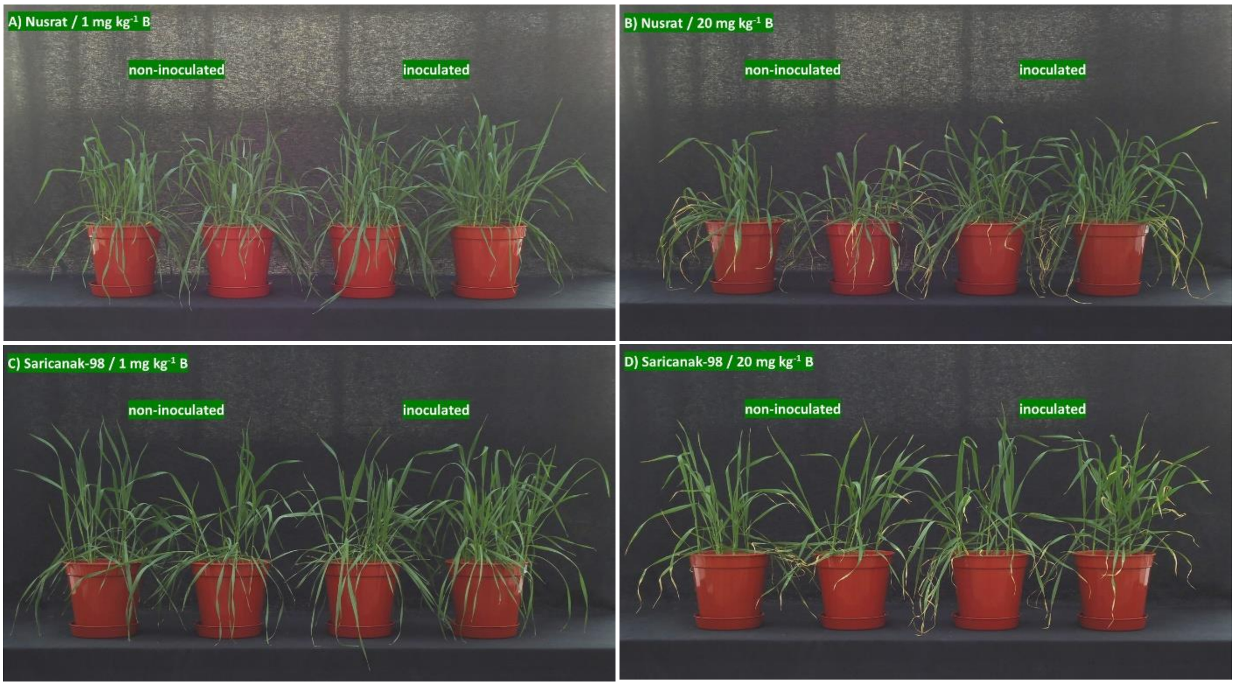

Figure S1: Effects of *P. indica* on 41-day-old bread wheat (*Triticum aestivum* cv. Nusrat) (A-B) and durum wheat (*Triticum durum* cv. Saricanak-98) (C-D) grown in non-autoclaved soil at control B (1 mg kg^{-1}) and medium B toxicity (20 mg kg^{-1}) levels under greenhouse conditions (Experiment 1).
