# Supplementary material for: The Fungal Root Endophyte Serendipita indica (Piriformospora indica) Enhances Bread and Durum Wheat Performance under Boron Toxicity at Both Vegetative and Generative Stages of Development through Mechanisms Unrelated to Mineral Homeostasis
Source: Biology (Basel). 2023 Aug 7;12(8):1098. doi: 10.3390/biology12081098 (PMC10452518; doi:10.3390/biology12081098)
Supplement: Supplementary file 1 [file biology-12-01098-s001.zip › Fig. S2.pdf]

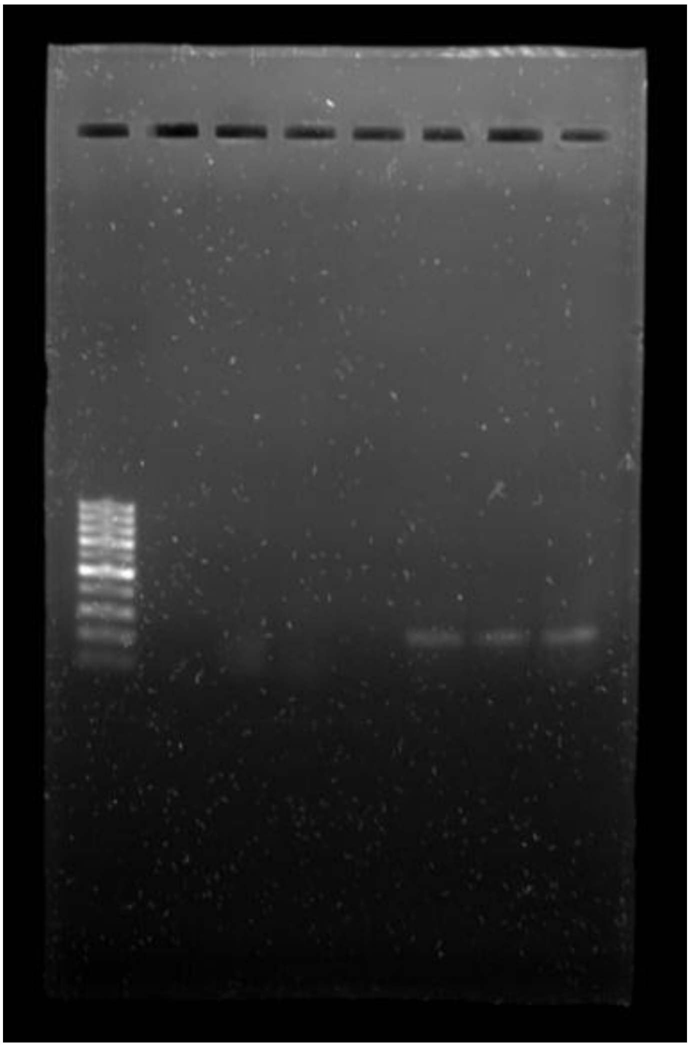

Figure S2: Original gel image - Polymerase chain reaction (PCR) detection of EF-1α (*tef*) gene of *S. indica* in DNA samples isolated from wheat roots 41 days after sowing.
